# Primary Cardiac Lymphoma: Importance of Tissue Diagnosis

**DOI:** 10.1155/2018/6192452

**Published:** 2018-07-25

**Authors:** Lauren Mendelson, Emily Hsu, Hojune Chung, Andrew Hsu

**Affiliations:** ^1^Department of Medicine, University of Massachusetts Medical School, Worcester, MA, USA; ^2^Department of Hematology and Oncology, Warren Alpert School of Medicine of Brown University, Providence, RI, USA

## Abstract

Primary cardiac lymphoma (PCL) is a rare disease entity that can present with severe cardiac and cardioembolic symptoms. We present a 79-year-old male with history of polymalgia rheumatica on chronic prednisone who presented with a two-week history of progressively worsening dyspnea, cough, and a 10 pound weight loss. Transthoracic echocardiogram (TTE) and computed tomography (CT) of the chest showed a large mediastinal mass with invasion of the pericardium. A biopsy of an abdominal soft-tissue mass confirmed the diagnosis of PCL. The patient was treated with two cycles of rituximab plus cyclophosphamide, doxorubicin, vincristine, and prednisone (R-CHOP) which was complicated by progressive heart failure requiring substitution of liposomal doxorubicin. The epidemiology, presentation, diagnosis, and treatment options of PCL are discussed.

## 1. Case Presentation

We present a 79-year-old male with history of polymyalgia rheumatica on chronic prednisone who presented with a two-week history of progressively worsening dyspnea, cough, and a 10 pound weight loss. He initially presented to an urgent care and had been prescribed antibiotics without improvement in his symptoms. He returned to the urgent care and underwent a chest X-ray, which was remarkable for cardiomegaly. Given this finding in conjunction with his respiratory symptoms, he was referred to the emergency department (ED).

**Figure 1:**
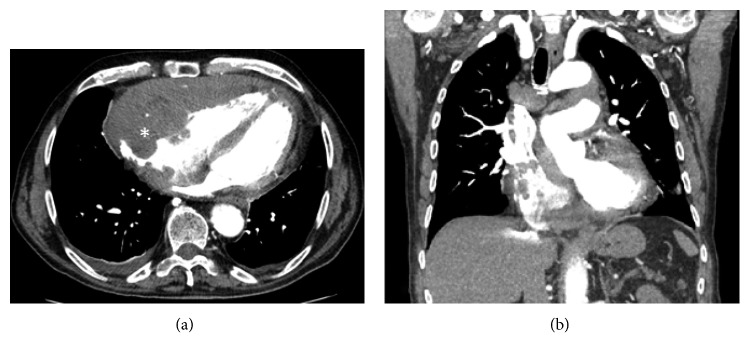
Computed tomography chest with pulmonary embolism protocol. (a) Transverse view and (b) coronal view. In the transverse view, there is extensive involvement of the anterior portion of the cardiac tissue and that encases the right atrium, right ventricle, and the great vessels (^∗^). There is evidence of filling defect in both the transverse and coronal views of the right atrium and ventricle, which suggests that the surrounding mass has infiltrated transmurally. The coronal view demonstrates how extensive the invasion is likely from an anterior to posterior approach. There is also extensive thickening of the transeptal and free wall.

In the ED, a CT of the chest showed a large mediastinal mass with invasion of the pericardium (Figure 1); a soft-tissue mass within the right atrium; compression of the left atrial appendage; encapsulation of the thoracic aorta and pulmonary artery; and extensive mediastinal, hilar, and subcarinal lymphadenopathy—the largest of which measured 3 cm in diameter. CT of the abdomen and pelvis showed numerous intra-abdominal and retroperitoneal soft-tissue masses.

**Figure 2:**
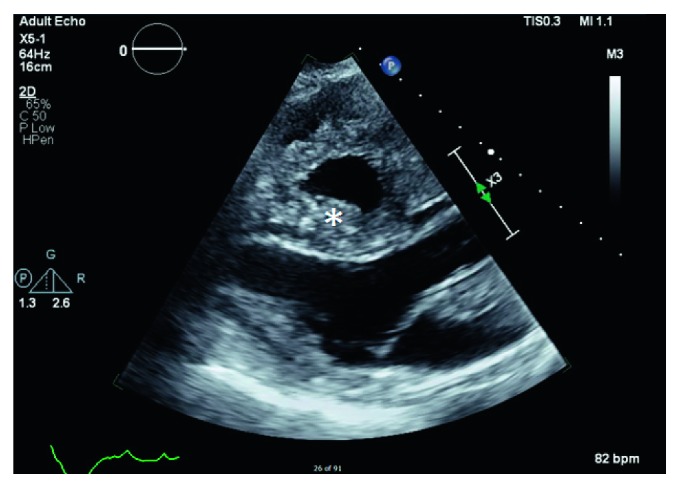
Transthoracic echo—parasternal long axis view. The cardiac cycle is in systole and the right ventricle fails to have concentric contraction demonstrated by the loss of curvature in the posterior right ventricle (^∗^). This indicates severe right ventricle dysfunction with severe hypokinesis/akinesis in the basal to apical wall due to infiltration. The cardiac tissue overall lacks any significant involvement of the posterior walls given the normal left atrium and left ventricle.

**Figure 3:**
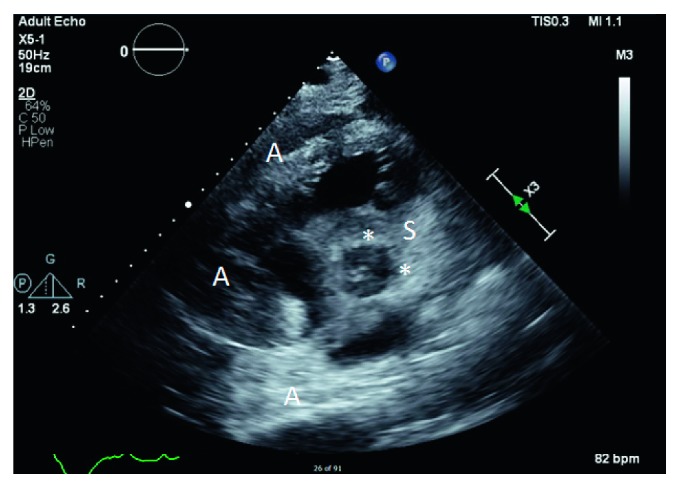
Transthoracic echo—parasternal short view. There is extensive homogenous echodense mass superiorly (S) and anteriorly (A) in relation to the cardiac tissue. The most striking feature is that despite the infiltration and surrounding of the cardiac structures of the right atrium, right ventricle, and the aortic valve, there is no evidence of tamponade physiology. The homogenous echodensity encases the ascending aorta at the level of the aortic valve (^∗^), masking the coronary ostia leading into the coronary arteries.

The patient was admitted to the intensive care unit (ICU) where a TTE showed a left ventricular ejection fracture of 55% along with a large, homogenous adherent mass infiltrating the right atrium and ventricle, abnormal thickening of the interatrial and interventricular septum of the right heart, severe right ventricular dysfunction, severe basal to apical hypokinesis to akinesis, and a pulmonary artery pressure of 21.8 mmHg (Figures 2 and 3).

Initial differential included primary lymphoma, cardiac sarcoma, or metastatic involvement of the heart. The patient underwent a biopsy of an abdominal soft-tissue mass. Pathology showed diffuse large B-cell lymphoma (DLBCL), nongerminal center type, with BCL2 and MYC.

The patient received intravenous (IV) methylprednisolone 250 mg daily for five days for debulking. He was initially treated with two cycles of R-CHOP; however, given his persistently reduced ejection fraction, the patient was changed to liposomal doxorubicin (R-CDOP) for the third and fourth cycle. Furthermore, his first cycle was also complicated by new onset first-degree atrioventricular block and a right bundle branch block. A positron emission tomography (PET)/CT scan and TTE were scheduled prior to the next cycle of R-CDOP. In addition to his systemic chemotherapy, the patient received three cycles of central nervous system prophylaxis with high-dose methotrexate.

## 2. Discussion

Malignancy of the heart is most often secondary to metastatic disease or direct invasion. Lymphoma, leukemia, and melanoma are the most frequent primaries that metastasize to the heart [1]. Primary cardiac tumors on the contrary are rare—200 were found in an autopsy series of 1,000,000 patients [2]. Within primary cardiac tumors, benign tumors are far more common than malignant tumors [3]. The most common benign cardiac tumors include myxoma, papillary fibroelastoma, and lipoma—these tumors account for almost 75% of all primary cardiac tumors. Malignant primary cardiac tumors are far less common and are primarily sarcomas. Far more rare malignant primary tumors include paragangliomas, extramedullary plasmacytomas, and primary lymphomas [1].

The World Health Organization (WHO) defines PCL as a lymphoma involving only the heart/pericardium or a lymphoma with the bulk of the tumor in the heart in the setting of clinical cardiac symptoms [4]. PCL accounts for 1.3% of primary cardiac tumors and 0.5% of extranodal lymphomas. Cardiac lymphoma can be either Hodgkin or non-Hodgkin B-cell lymphoma but are most commonly DLBCL. PCL is more common in the immunosuppressed patient (AIDS, post-transplant) secondary to Epstein–Barr virus-related lymphoproliferation [5].

Primary cardiac tumors including PCL do not have a pathognomonic presentation, rather they present based on their location in the heart. Right-sided tumors present with signs and symptoms of right-sided heart failure if they are obstructing blood flow, or they can present with symptoms of pulmonary emboli from embolic tumor fragments into the lungs. Left-sided tumors can present with signs and symptoms of left-sided heart failure if they are obstructing blood flow, or they can present as an ischemic stroke from embolic tumor into the CNS. Lastly, left ventricular tumors that are intramural can present with arrhythmias or conduction defects. An institutional study at the Mayo Clinic found the most common patient complaint on presentation to be dyspnea on exertion (79%) followed by nonspecific chest pain (38%) and cough (21%) [6].

The diagnosis of primary cardiac tumors is based upon imaging and biopsy findings. TTE is often the initial image modality but is limited by operator-expertise and body habitus. CT of the chest can be used but is limited in soft-tissue contrast. Cardiac magnetic resonance imaging (MRI) is the preferred imaging modality, as it provides an unrestricted view, high temporal resolution, and good soft-tissue contrast to help characterize a cardiac mass. In PCL, the tumor often appears as a large nodular mass that is isoattenuating to myocardium on CT, isointense to myocardium on T1, and hyperintense to myocardium on T2 [7].

Imaging often greatly helps characterize the type of primary cardiac tumor. Often the differentiation between benign and malignant or even the specific disease diagnosis can be made based upon imaging alone. Depending on the imaging and suspected etiology of malignancy, tissue sampling may or may not be warranted. If imaging cannot characterize the mass, a discussion of the risks and benefits of an invasive biopsy must take place. Methods of obtaining a tissue diagnosis include myocardial biopsy during exploratory thoracotomy, pericardiocentesis if pericardial effusion present, TEE-guided biopsy, mediastinoscopy, and endomyocardial transvenous biopsy [8]. In our case, it was imperative to obtain a tissue diagnosis with malignant cardiac tumor on the differential. Tissue was obtained from an abdominal mass, which had a lower complication rate than the procedures listed above.

Primary cardiac tumors are treated differently based on the specific pathologic disease. There is no gold standard of treatment for PCL. In reported cases, anthracycline-based chemotherapy and rituximab was associated with prolonged survival [9, 10]. There is a limited role for surgery in PCL, unlike many other cardiac tumors. There is utility in surgical resection if the tumor causes life-threatening hemodynamic compromise; however, there is no evidence of prolonged survival with surgery alone or combined with chemotherapy in a hemodynamically stable patient [11]. It is unclear based on a small number of cases whether radiotherapy combined with chemotherapy is superior to chemotherapy alone; furthermore, the risk of cardiopulmonary radiation-induced injury makes it a less preferred treatment modality [9, 10, 12]. The median survival ranges from 1.5–26.5 months [13]. Our patient was treated with two cycles of R-CHOP which was complicated by progressive heart failure requiring substitution of liposomal doxorubicin.

## 3. Conclusion

In conclusion, PCL is a rare disease that accounts for 1.3% of primary cardiac tumors and 0.5% of extranodal lymphomas. The disease does not have a pathognomonic presentation, rather it presents based on its location in the heart with signs of heart failure or cardioembolic phenomena. PCL is diagnosed based on imaging and tissue biopsy. If there is a high suspicion for PCL based on imaging, it is important to obtain a tissue biopsy. Definitive tissue diagnosis of PCL can then be treated with anthracycline-based chemotherapy plus rituximab, which has been associated with prolonged survival.

## References

[1] Neragi-Miandoab S., Kim J., Vlahakes G. J. (2007). Malignant tumours of the heart: a review of tumour type, diagnosis and therapy. *Clinical Oncology*.

[2] Reynen K. (1996). Frequency of primary tumors of the heart. *American Journal of Cardiology*.

[3] Vander Salm T. J. (2000). Unusual primary tumors of the heart. *Seminars in Thoracic and Cardiovascular Surgery*.

[4] Burke A., Tavora F. (2016). The 2015 WHO classification of tumors of the heart and pericardium. *Journal of Thoracic Oncology*.

[5] Jeudy J., Kirsch J., Tavora F. (2012). From the radiologic pathology archives: cardiac lymphoma: radiologic-pathologic correlation. *Radiographics*.

[6] Simpson L., Kumar S. K., Okuno S. H. (2008). Malignant primary cardiac tumors: review of a single institution experience. *Cancer*.

[7] Hoey E. T., Mankad K., Puppala S., Gopalan D., Sivananthan M. U. (2009). MRI and CT appearances of cardiac tumours in adults. *Clinical Radiology*.

[8] Ceresoli G. L., Ferreri A. J., Bucci E. (1997). Primary cardiac lymphoma in immunocompetent patients: diagnostic and therapeutic management. *Cancer*.

[9] Dawson M. A., Mariani J., Taylor A., Koulouris G., Avery S. (2006). The successful treatment of primary cardiac lymphoma with a dose-dense schedule of rituximab plus CHOP. *Annals of Oncology*.

[10] Shin D.-Y., Lee Y.-G., Lee H.-J., Choi S., Park J. J., Kim D.-W. (2010). Long-term disease-free survival of patients with primary cardiac lymphoma treated with systemic chemotherapy and radiotherapy. *Korean Journal of Hematology*.

[11] Gosev I., Sirić F., Gasparović H. (2006). Surgical treatment of a primary cardiac lymphoma presenting with tamponade physiology. *Journal of Cardiac Surgery*.

[12] Madan R., Benson R., Sharma D. N., Julka P. K., Rath G. K. (2015). Radiation induced heart disease: pathogenesis, management and review literature. *Journal of the Egyptian National Cancer Institute*.

[13] Anghel G., Zoli V., Petti N. (2004). Primary cardiac lymphoma: report of two cases occurring in immunocompetent subjects. *Leukemia and Lymphoma*.

